# High immunocompetence in chronic hepatitis patients with normal alanine transaminase levels and and negative hepatitis B e‐antigen for the progression of liver fibrosis

**DOI:** 10.1002/iid3.1134

**Published:** 2024-01-17

**Authors:** Bianqiao Cheng, Dandan Wu, Ming Zhang, Shiming Chen, Xunyuan Wu, Jingjing Zhong, Meimei Wu, Miaomiao Huo

**Affiliations:** ^1^ Department of Gastroenterology Fuzhou Second Hospital Fujian China; ^2^ Department of Clinical Medicine Fujian Medical University Fujian China; ^3^ Department of Infection Management Fuzhou Second Hospital Fujian China

**Keywords:** ALT, CHB, HBeAg‐negative, progressive liver fibrosis, Treg

## Abstract

**Introduction:**

This study aimed to investigate the role of immunocompetence in chronic hepatitis B (CHB) patients with normal alanine transaminase (ALT) levels and negative hepatitis B e antigen (HBeAg) in the risk assessments of the progression of liver fibrosis.

**Methods:**

We collected the clinical data of 57 patients with CHB, with normal ALT levels and negative HBeAg from December 2020 to December 2022. With hepatitis B virus (HBV) DNA > 20 IU/mL and ALT ≤ 40 U/L, these patients had never undergone antiviral therapy. The levels of CD4^+^, CD4^+^CD25^+^, CD8^+^, and CD4^+^CD25^+^CD127^LOW^ regulatory T cells (Tregs) in the patients were detected using flow cytometry; the liver stiffness measurement (LSM) values of the patients were detected using Fibroscan.

**Results:**

There was a statistically significant difference between the levels of fibrosis‐4 (FIB‐4) and hepatitis B surface antigen (HBsAg) when the cutoff point was HBsAg ≥ 1500 (*p* < .001). FIB‐4 was negatively correlated with HBsAg (*R* = −0.291, *p* = .028) and positively correlated with age (*R* = 0.787, *p* < .001). LSM was negatively correlated with Treg but this correlation was not statistically significant (*p* > .05). Findings based on the analysis using logistic regression were as follows: (i) age was the independent risk factor when FIB‐4 was used as the indicator for assessing liver fibrosis; (ii) Treg was the independent risk factor when LSM was used as the indicator for assessing liver fibrosis. When Treg was used to predict liver fibrosis, the cutoff value, diagnostic efficacy, area under the receiver operating characteristic (ROC) curve, and *p* value of the ROC curve were 6.875, 0.641, 0.84, and .027, respectively.

**Conclusion:**

Age and Treg are independent risk factors for progressive liver fibrosis. The cutoff value of Treg > 6.81 indicates the need for timely antiviral treatment and can serve as an indicator for evaluating liver fibrosis.

## Graphical abstract

This study aimed to investigate the role of immunocompetence in chronic hepatitis B (CHB) patients with normal alanine transaminase (ALT) levels and negative hepatitis B e antigen (HBeAg) in the risk assessments of the progression of liver fibrosis. Age and Treg are independent risk factors for progressive liver fibrosis. Treg ≤ 6.875 indicates the need for timely antiviral treatment and can serve as an indicator for evaluating liver fibrosis.

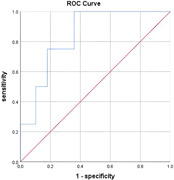

## 1 INTRODUCTION

Currently, there are 350 million hepatitis B virus (HBV) carriers worldwide, with 120 million in our country, and ~300,000 deaths per year results from liver cirrhosis and liver cancer caused by hepatitis B virus (HBV) infection.^1^, ^2^, ^3^ Due to the asymptomatic and normal liver function status of HBV carriers, the awareness of the harm from HBV to the human body is deficient and timely antiviral treatment is not initiated, resulting in a continuous increase in the incidence of liver cancer triggered by HBV, threatening to human health. Timely antiviral treatment for HBV can significantly reduce the occurrence of liver cirrhosis, liver cancer, and other complications.

The latest chronic hepatitis B diagnosis and treatment guidelines released by the American Association for the Study of Liver Diseases, the European Association for the Study of the Liver, and the Asian Pacific Association for the Study of the Liver^4^, ^5^, ^6^ divide HBV infection into four phases as follows: (1) immune tolerance phase; (2) immune active or immune clearance phase; (3) inactive or low‐replication phase; and (4) reactivation phase. Clinically, normal alanine transaminase (ALT) levels in HBV‐infected person often indicate the immune tolerance or immune control phase of chronic hepatitis B and a spontaneous transition from immune tolerance to immune control occurs in a certain percentage of patients each year.^7^ During this phase, patients typically have negative serum hepatitis B e antigen (HBeAg), undetectable or low levels of HBV DNA (<2000 IU/mL), low levels of serum HBsAg (<1500 IU/mL), and sustained normal ALT levels with mild liver inflammation and minimal fibrosis. Patients in this phase generally have no disease progression, a low incidence of adverse events, and some patients may experience spontaneous seroconversion of HBsAg. Therefore, it is recommended that the relative treatment is not necessary.^8^, ^9^, ^10^ However, some experts have proposed that age, HBsAg levels, and HBV DNA levels become risk factors for the progression of liver fibrosis and liver cancer in the immune control phase as they age and the disease progresses.^11^, ^12^, ^13^, ^14^

The indications for antiviral treatment of chronic hepatitis B in domestic guidelines have been gradually relaxed in 2022,^15^, ^16^ but there are still a considerable number of patients with chronic hepatitis B, who do not meet the current indications and are unable to receive antiviral treatment. In particular, recent studies have found that a proportion of HBV carriers with persistently normal ALT levels progress to liver cirrhosis and liver cancer within 5–10 years.^17^, ^18^ Therefore, for patients who do not meet the current guidelines for antiviral treatment, failure to provide timely antiviral treatment may result in the occurrence of hepatocellular carcinoma (HCC) or disease progression leading to death.

Multiple studies have shown that even when ALT is within the upper limit of normal range, there is a correlation with the occurrence of liver fibrosis.^19^, ^20^, ^21^, ^22^ Some researchers have found that 40%–70% of patients with chronic HBV infection have persistently normal ALT levels,^23^, ^24^ but liver histology indicates the presence of inflammation or fibrosis, and some even progress silently to liver cirrhosis and liver cancer. Early identification and timely antiviral intervention in patients with chronic HBV infection can significantly delay disease progression and improve prognosis.^6^, ^7^

There is currently no consensus on the timing of antiviral treatment for patients with chronic hepatitis B and normal ALT levels. Both domestic and international guidelines recommend that patients who are not eligible for antiviral treatment should undergo liver biopsy to determine the severity of the disease.^15^, ^16^ However, due to its invasive feature, potential errors in pathological testing, and patient acceptance issues, it can not be widely applied in clinic. Liver transient elastography (liver stiffness measurement [LSM]) and the fibrosis 4 score (FIB‐4) are internationally recognized methods for noninvasive assessment of liver fibrosis in patients with chronic liver disease. The combination of the two can be used as an effective evaluation index for liver fibrosis, replacing liver biopsy. For patients with normal liver function and LSM > 7.4 or FIB‐4 > 1.45, there is an indication of liver fibrosis; Stage 2 or above fibrosis have a 94.7% concordance rate with liver bioptic results.^25^

In the chronic infection process of HBV, the host's immune response determines the ultimate outcome. Regulatory T cells (Treg) play a role in suppressing the proliferation and differentiation of HBV‐specific CD8^+^ T cells and the antiviral effect on Th1‐type cells, leading to immune tolerance. Therefore, the level of Treg content plays a regulatory role in the host's antiviral response to HBV. Studies by Zhou et al.^26^, ^27^ found that Treg cells are slightly increased in the immune tolerance and inactive phases compared with normal individuals. However, in the immune activation period, Treg cells were increased significantly, and fibrosis and inflammation accelerated gradually. This suggests that the assessment of Treg level in HBV host can understand the immune response state of the body and has a reference value for the selection of HBV antiviral treatment time.

Thus, this study collected clinical and immunological indicators of chronic hepatitis B (CHB) patients with normal ALT and negative HBeAg, and analyzed the correlation between Treg cell level and liver fibrosis indicators FIB‐4 and LSM, to explore the risk factors causing liver fibrosis and to evaluate the risk coefficient and clinical efficacy. It is expected to provide a clear and accurate reference standard for the evaluation of antiviral therapy and disease development in CHB patients.

## 2 METHODS

### 2.1 Participant selection and data collection

A total of 98 HBeAg‐negative CHB patients from Fuzhou Second Hospital in Fujian Province, China, from December 2020 to December 2022, were enrolled in this study. This research was approved by the Fuzhou Second Hospital Ethics Committee (Approved Number 2020009) Guidelines for the Prevention and Treatment of Chronic Hepatitis B 2019 and 2022.^28^, ^29^ The selection criteria were as follows: (1) Serum HBsAg positivity for more than 6 months, patients who were HBeAg‐negative, and ALT < 40 U/L. (2) Patients with CHB, who had never received therapy. (3) Patients with CHB, who were willing to participate in the research and signed an informed consent form. The exclusion criteria were as follows: (1) Patients with CHB whose liver dysfunction was caused by factors other than HBV, such as other viruses, alcohol, drugs, or a combination of these factors. (2) Patients with CHB whose ALT levels exceeded the normal upper limit. (3) HBV carriers who were receiving antiviral treatment. (4) Patients with CHB, who were unable to cooperate in relevant examinations due to severe heart, liver, kidney or endocrine disorders, hematopoiesis, or psychoneurosis. (5) Pregnant or nursing mothers. (6) Patients with CHB, who did not cooperate or who dropped out of the study. (7) Patients for whom we lack sufficient information to meet our statistical requirements.

The gender, age, blood routine, liver function, five liver indexes (HBsAg, anti‐HBs [hepatitis B surface antibody], HBeAg, anti‐HBe, and anti‐HBc [hepatitis B core antibody]), HBV DNA, and FIB‐4 were collected from all participants.

### 2.2 Liver fibrosis detection

FIB‐4 could be used as a reference index for the diagnosis and staging of liver fibrosis in CHB patients, and it was correlated with ALT. Aspartate aminotransferase, platelet count (PLT), and AST are age‐related. FIB‐4 was calculated as: (age × AST)/(PLT × √ALT). FIB‐4 < 1.45 excluded advanced hepatic fibrosis.

LSM values of the patients were detected using Fibroscan, a transient elastography (TE) scanner (Fibroscan 502 from Echosens). According to the Consensus on Diagnosis and Treatment of Hepatic Fibrosis in 2019^30^ jointly developed by the Divisions of Hepatology, Gastroenterology, and Infectious Disease of the Chinese Medical Association, we determined that patients were not in the progressive stage of liver fibrosis when their TE was <7.4 kPa.

#### 2.2.1 Flow cytometry

The steps were as follows according to the operation instruction: (1) Four assay tubes were labeled A as blank control, B as peer control for CD4^+^ and CD25^+^, C as test group for CD4^+^ and CD25^+^, and D as test group for CD8^+^; 0.2 mL whole blood pretreated with EDTA and 2 mL red cell cracking fluid were added in each assay tube, then they were shaken and mixed, incubated at 4°C for 20 min in the dark. (2) They were centrifuged at 800*g* for 5 min and the supernatant was discarded. (3) 1 mL phosphate‐buffered saline (PBS) was added in each tube, then centrifuged and resuspend twice, and the supernatant was discarded. (4) 0.2 mL PBS was added in tube A; in tube B was added 0.2 mL PBS, PerCP‐CD4, and PE‐CD25 homologous control with 40 μL; PerCP‐CD 40 μL, PE‐CD25 40 μL, and Alexa 647‐CD127 30 μL were added into the C tube; PE‐CD8 30 μL was added into the D tube (the optimal dose of antibody was combined with the reagent instructions and the pre‐experimental titration results), then incubation at 4°C for 30 min. (5) 0.8 mL PBS was added in each tube and the supernatant was repeatedly centrifuged, suspended, and abandoned twice. (6) The peripheral blood was then resuspended in 0.5 mL of PBS and tested using a flow cytometry device (Device specification: BD FACSVerse, BD). (7) Each tube was tested using a flow cytometry device (Device specification: BD FACSVerse, BD).

#### 2.2.2 Statistical analysis

SPSS26.0 was used for the statistical description and analysis of the data. The values for normally distributed and non‐normally distributed measurement data were expressed as mean ± SD and median (Q1, Q3), respectively, in description of baseline features and correlation factor analysis. Enumeration data were expressed as (*n* [%]). Paired sample *T* test and Mann–Whitney *U* test (or Wilcoxon rank‐sum test) were utilized for normally and non‐normally distributed measurement data after comparing data between groups, respectively. After comparing enumeration data between groups, the *χ* ^2^ test was applied to the results. Spearman's correlation was utilized for the correlation analysis of liver fibrosis factors. Using logistic regression, the risk factors for progressive liver fibrosis were analyzed in risk factor identification. The receiver operating characteristic (ROC) curve was utilized to evaluate the diagnostic performance of risk factors in diagnostic effectiveness evaluation. *p* < .05 indicated a statistically significant difference.

## 3 RESULTS

### 3.1 Baseline characteristics

A total of 99 HBeAg‐negative CHB patients with normal ALT levels were enrolled. Forty‐one cases were excluded due to HBV DNA < 20 IU/mL, ALT ≥ 2 × ULN, and incomplete data; finally, a total of 57 cases were enrolled in this study. The baseline characteristics of the included patients were shown in Table 1. The 57 participants included 39 men and 18 women, whose minimum and maximum ages were 20 and 74 years, respectively, with an average age of 47.79 ± 14.26 years. Patients with HBsAg < 1500 IU/mL, HBV DNA < 2000 IU/mL, FIB‐4 ≥ 1.45, and LSM ≥ 7.4 accounted for 54 (94.7%), 31 (54%), 19 (33.3%), and 5 (8.7%), respectively. The cutoff of HBsAg was 1500 IU/mL. There was a statistical difference between the levels of FIB‐4 and HBsAg (*p* < .001). However, age, gender, LSM, platelet levels, HBV DNA level, CD4^+^, CD8^+^, and Treg level were not statistically significant among patients with HBsAg < 1500 IU/mL and HBsAg ≥ 1500 IU/mL.

**Table 1:** Baseline characterization.

Variable	HBsAg＜1500 (*n* = 54)	HBsAg ≥ 1500 (*n* = 3)	*p*
Gender			
Male	37	2	.946
Female	17	1	
Age	52.94 ± 16.573	39 ± 9.539	.157
FIB‐4	1.1588 (0.8259, 1.658)	0.633 (0.5314, 0.7468)	.032
LSM	5.05 (4.2, 6.05)	4.3 (4.1, 6)	.567
HBV DNA	1164.5 (371.725, 4881)	7632 (197.8, 10260)	.617
PLT	206 (177, 243.5)	209 (186, 263)	.716
ALT	18 (13, 23)	20 (14, 21)	.9
CD4^+^	30.4676 ± 9.31166	31.5333 ± 14.96841	.852
CD4^+^CD25^+^/CD4^+^	8.2106 ± 2.85601	7.53 ± 3.42869	.692
CD4^+^CD25^+^CD127^−^/CD4^+^ (Treg)	6.7852 ± 2.77927	6.36 ± 2.22	.881
CD8^+^	18.7647 ± 6.78355	16.2667 ± 5.60744	.535

Abbreviations: ALT, alanine transaminase; FIB‐4, fibrosis‐4; HBsAg, hepatitis B surface antigen; HBV, hepatitis B virus; LSM, liver stiffness measurement; PLT, platelet.

### 3.2 Baseline characteristics of liver fibrosis

FIB‐4 and LSM are effective noninvasive indexes for evaluating liver fibrosis, except for liver biopsy, and the combination of FIB‐4 and LSM is more effective for evaluating liver fibrosis. We used FIB‐4 and LSM values as live fibrosis reference indicators.^30^ As shown in Table 2, the cutoff of FIB‐4 was 1.45 and the statistically significant differences in age (*p* < .001) and HBsAg (*p* = .023) between the FIB‐4 groups, which result hinted age and HBsAg level, were certain correlation with the progression of liver fibrosis on FIB‐4. When the cutoff of LSM was 7.4, there was a statistically difference in Treg between groups based (*p* = .046), which hinted Treg as an indicator of hepatic fibrosis progression. These results above indicated that with the increase of age and the increase of HBsAg level, Treg and immunosuppressive state were positively correlated with the occurrence of liver fibrosis.

**Table 2:** Analysis of indicators related to liver fibrosis.

Variable		Full sample (*n* = 57)	FIB‐4			LSM		*H*
			≤1.45 (*n* = 38)	＞1.45 (*n* = 19)	*p*	LSM ≤ 7.4 (*n* = 52)	LSM＞7.4 (*n* = 5)	*p*
Gender								
Male		39 (68.4%)	28 (26.3%)	8 (42.1%)	.23	35 (67.3%)	4 (80%)	.57
Female		18 (31.6%)	10 (73.7%)	11 (57.9%)		17 (32.7%)	1 (20%)	
Age		52.21 ± 12.25	44.82 ± 12.92	67 ± 12.49	0*	50.87 ± 16.23	66.2 ± 13.29	.04*
HBV DNA		1218 (359, 5959)	1839 (399,6659)	918.9 (294, 5583)	.66	1322.5 (347, 5913)	497.2 (339, 15616)	.71
PLT		206 (177, 243)	219 (183, 258)	192 (169, 202)	.03*	206 (179, 243)	199 (153, 269)	.84
HBsAg		128.52 (23, 250)	250 (41.2, 324.8)	42.2 (9.7, 250)	.02*	189.26 (18.73, 250)	113.39 (21.755, 250)	.42
ALT		18 (13, 22.5)	18.5 (14, 23.25)	15 (12, 21)	.12	18.5 (14, 23)	14 (6.5, 20)	.07
CD4^+^		30.3 (24, 37.95)	30.1 (23.2, 37.875)	31.5 (24.7, 38.7)	.72	30.1 (24.55, 37.6)	32.7 (15.275, 43)	.87
CD4^+^CD25^+^		7.8 (6.2, 10.05)	8.035 (6.13, 10.37)	7.28 (6.13, 8.63)	.71	7.59 (6.085, 9.88)	8.63 (7.75, 11.5)	.31
Treg		6.36 (5.07, 7.95)	5.855 (4.96, 7.84)	6.89 (5.3, 8.65)	.32	5.86 (5.03, 7.82)	8.575 (7.16, 14.4225)	.01*
CD8^+^		17.7 (12.4, 23.6)	17.15 (12.17, 23.52)	20.2 (13.5, 24.9)	.49	17.6 (12.2, 23.6)	20.2 (13.465, 24.55)	.83

*Note*: *χ*2 test was used for gender comparison of count data. The *t* test was used to determine the normal distribution of age. If HBV DNA, PLT, HBsAg, ALT, CD4 ^+^ , CD4 ^+^ CD25^+^ , Treg, and CD8^+^ did not meet the normal distribution, Mann–Whitney *U* rank‐sum test was used.

Abbreviations: ALT, alanine transaminase; FIB‐4, fibrosis‐4; HBsAg, hepatitis B surface antigen; HBV, hepatitis B virus; LSM, liver stiffness measurement; PLT, platelet.

* *p* < 0.05 difference was statistically significant.

### 3.3 Factors associated with liver fibrosis

According to the statistical results in Table 2, it was preliminarily confirmed that age, HBsAg level, and Treg were related to liver fibrosis. Here we further discussed the correlation between the above indicators with LSM and FIB‐4. FIB‐4 had a positive correlation with age (*R* = 0.783) but a negative correlation with HBsAg levels (*R* = −0.291) by Spearman's correlation. LSM correlated positively with age (*R* = 0.226) and negatively with Treg (*R* = −0.05) (Table 3). These results indicated that liver fibrosis might progress as Treg levels and age increase.

**Table 3:** Spearman correlation analysis was used to analyze the related factors of liver fibrosis.

Variable	FIB‐4		LSM (Kpa)	
	*r*	*p*	*r*	*p*
Age	.783	0*	.226	.091
Treg	–	–	−.05	.752
PLT	−.415	.001*	–	–
HBsAg	−.291	.028*	–	–

Abbreviations: FIB‐4, fibrosis‐4; HBsAg, hepatitis B surface antigen; LSM, liver stiffness measurement; PLT, platelet; Treg, regulatory T cell.

* *p* < .05.

### 3.4 Risk factor identification

Based on the above results, we hypothesized that the improvement of immunocompetence correlated with the progression of liver fibrosis. We used logistic regression to further investigate the risk factors for progressive liver fibrosis. The group with FIB‐4 > 1.45 had an increased risk of liver fibrosis compared with the group with FIB‐4 < 1.45 (odds ratio [OR] = 1.416, 95% confidence interval [95% CI]: 1.026–1.953), as shown in Table 4. When the frequency of Treg was >7.4 compared with <7.4 for LSM, the risk of fibrosis was increased (OR = 1.416, 95% CI: 1.026–1.953), as shown in Table 5. Age and Treg correlated significantly with liver fibrosis; they could serve as risk factors for liver fibrosis progression.

**Table 4:** Logistic analysis showed that LSM 7.4 kPa was a risk factor for liver fibrosis.

Variable	Univariate logistic regression		Multivariate logistic regression	
	OR	*p*	OR	*p*
Age	1.061 (0.996 ~ 1.13)	.064		
Treg	1.416 (1.026 ~ 1.953)	.034*	1.496 (1.003 ~ 2.231)	.048*

Abbreviations: LSM, liver stiffness measurement; OR, odds ratio; Treg, regulatory T cell.

* *p* < .05.

**Table 5:** Risk factors of liver fibrosis predicted by FIB‐4 > 1.45 were analyzed by logistic regression.

Variable	Univariate logistic regression		Multivariate logistic regression	
	HR (95% CI)	*p*	HR (95% CI)	*p*
Age	1.129 (1.061 ~ 1.2)	0*	1.272 (1.098 ~ 1.473)	.001*
PLT	0.985 (0.971 ~ 1)	.043*	0.957 (0.922 ~ 0.993)	.018*
HBsAg	0.998 (0.996 ~ 1.001)	.135		

Abbreviations: CI, confidence interval; FIB‐4, fibrosis‐4; HR, hazard ratio; PLT, platelet.

* *p* < .05.

### 3.5 Diagnostic efficiency evaluation

**Figure 1:**
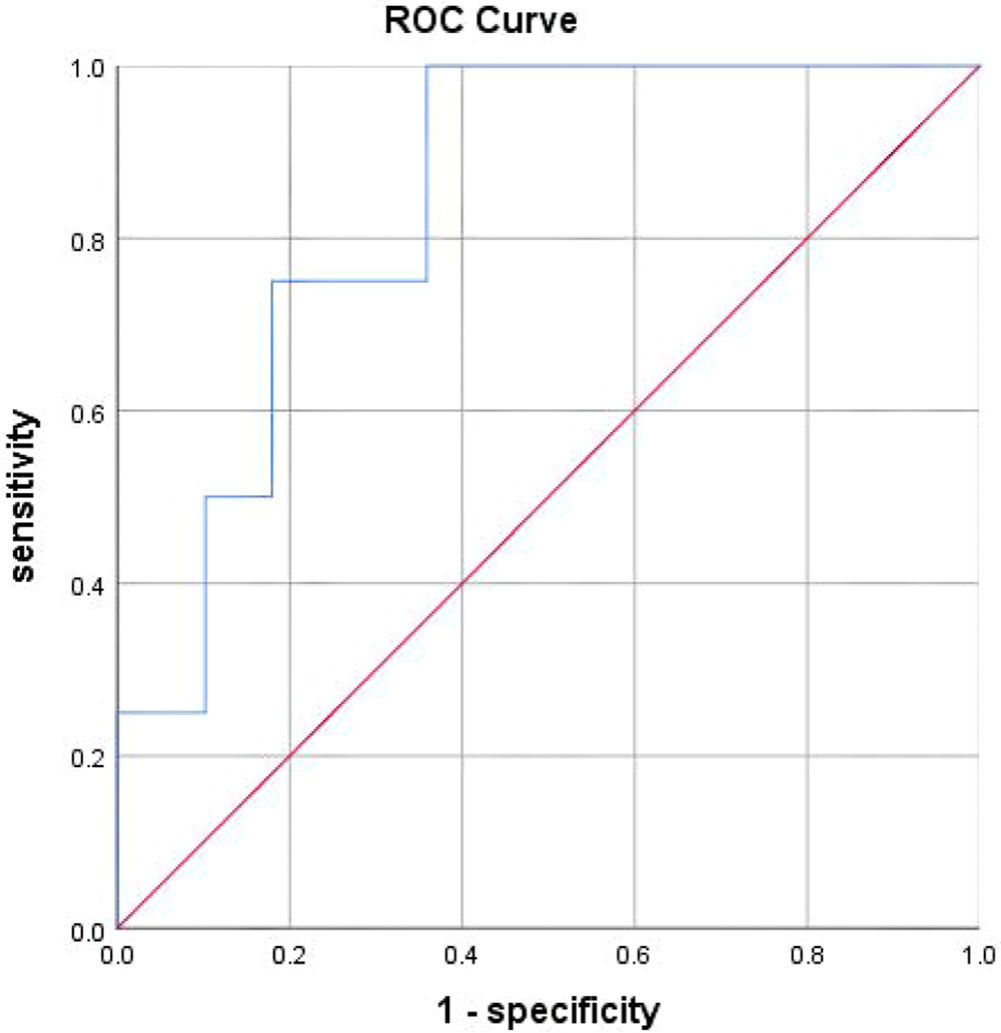
Receiver operating characteristic (ROC) curve to evaluate the diagnostic efficacy of regulatory T cell (Treg) as a predictor of liver fibrosis. When the cutoff of Treg cells was 6.875, the area under the ROC curve for predicting liver fibrosis was 0.84, the sensitivity of true positive rate was 75.0%, the specificity of true negative rate was 64.1%, and the *p* value was 0.027.

According to Logistic regression analysis, it was found that age and Treg were risk factors for liver fibrosis. Then, age and Treg were used as variables to evaluate their efficacy by ROC curve. The results showed that when the age was more than 57 years and the cutoff of Treg level was 6.81, the area under the ROC curve (95% CI) for the diagnosis of advanced liver fibrosis was 0.84 (0.685 ~ 0.995), the specificity was 64.1%, and the sensitivity was 64.1%, *p* = .027. The results were shown in Figure 1, which indicated Treg level > 6.81 as a potential risk of developing liver fibrosis and the need for prompt antiviral treatment.

## 4 DISCUSSION

HBVcccDNA is a covalent, closed, circular DNA molecule that forms supercoiled structures in host liver cells upon HBV infection. It is responsible for continuous viral replication, resulting in chronic inflammation, fibroproliferation, activation of cancer‐related genes, and impaired immune defense mechanisms in the liver. Ultimately, this can lead to the development of end‐stage liver diseases, such as liver cirrhosis and HCC. Antiviral therapy for HBV can significantly reduce the incidence of end‐stage liver diseases.

Regarding the treatment of chronic HBV, there is currently no clear consensus on whether antiviral therapy is necessary for patients in the “immune tolerance phase” or the “unclassified phase.”^31^ Specifically, for patients with normal ALT levels, low HBV DNA levels, and negative HBeAg, some experts believe that the disease is stable, and that treatment is unnecessary. In fact, it is important to note that having “normal” liver function does not necessarily mean the absence of inflammation in the liver.^32^ In this study, we found that among CHB patients with normal ALT levels, the proportion of liver fibrosis was 33.3% (19/57 cases) (using FIB‐4 > 1.45 as an indicator of liver fibrosis) or 8.7% (5/57 cases) (LSM > 7.4 as an indicator of progressive liver fibrosis). The discrepancy stems from not considering abnormal values of ALT and bilirubin when evaluating liver fibrosis using LSM. Nevertheless, the results suggested that HBeAg‐negative CHB patients with normal ALT levels still carry a risk of disease progression.

Immunologic tolerance‐CHB (IT‐CHB) transitioning into the immune clearance phase often occurs during the third decade of life, with a considerable proportion (22.5%–34%) of IT‐CHB patients exhibiting liver fibrosis. Those studies indicated that fibrosis progression may have already begun during the immune tolerance phase or at an earlier stage. Liver fibrosis becomes more prevalent with increasing age, suggesting a disruption of immune tolerance mechanisms and the transition of HBV infection into an immune activation phase.^33^ Age, therefore, becomes a risk factor for liver fibrosis. Regardless of HBeAg positivity, patients over 40 years old exhibit significant liver damage in more than half of the cases.^34^ Compared with patients under the age of 30 years, CHB patients over 40 years old are more prone to develop liver cirrhosis and HCC.^35^ The 2022 Chinese Guidelines for Chronic Hepatitis B^16^ suggest paying more attention to HBeAg‐negative chronic HBV‐infected patients with normal ALT levels and age ≤ 30 years.^17^, ^24^ This study found a positive correlation between age and liver fibrosis, indicating a significantly higher risk of developing liver fibrosis in the liver fibrosis group compared with the nonfibrosis group when the age reaches 57 years. This finding aligns with the results of this study, further supporting age as a risk factor in predicting liver fibrosis.

Immune activity is a major factor in the progression and chronicity of HBV infection. Treg cells, a subpopulation of T cells involved in controlling self‐immune responses, exhibit significantly increased tendency during active HBV infection. This study found that the Treg cell count was higher in the liver fibrosis group than in the nonfibrosis group, with a significant difference between the two groups. The count of Tregs was positively correlated with progressive liver fibrosis, and when the threshold value was set at 6.81, the risk of developing liver fibrosis was significantly higher in the progressive fibrosis group compared with that in the nonfibrosis group (*p* = .027). However, the difference between CD4^+^ and CD8^+^ lymphocytes were not significant in the liver fibrosis group and the nonfibrosis group. This finding is consistent with previous research,^25^ which suggests that Treg cell levels are significantly higher during the immune clearance phase compared to immune tolerance and inactive phases. Zhou et al.^26^ concluded that Treg content in immune clearance stage was significantly higher than that in immune tolerance stage and inactive stage, and inflammatory response and liver fibrosis were positively correlated with Treg content.^27^ Therefore, the value of Tregs cell in guiding antiviral screening in CHB patients should be emphasized.

Due to the limited sample size in this study, it may affect the predictive value of Tregs cell in assessing liver fibrosis. According to a threshold value (<5% for Tregs) in healthy individuals,^36^ our data indicated that Treg values >6.81 still have vitally diagnostic value for progressive liver fibrosis in HBV patients. Next, a new research in multiple centers, containing more samples, will be conducted to pinpoint the threshold value of Treg in the risk assessment of liver fibrosis progression. Meanwhile, the diagnostic efficacy of Treg in patients with CHB is further verified by applying Treg predictive value in clinical practice, which could offer a meaningful strategy for antiviral treatment in HBeAg‐negative patients with CHB with normal ALT.

## 5 CONCLUSION

Age and Treg were independent risk factors for progressive liver fibrosis. The cutoff value of Treg > 6.81 indicated the need for timely antiviral treatment and could serve as an indicator for evaluating liver fibrosis.

## AUTHOR CONTRIBUTION

**Bianqiao Cheng**: Conceptualization, writing—original draft, critical revision, funding acquisition. **Dandan Wu**: Conceptualization, data curation, formal analysis, critical revision. **Ming Zhang**: Conceptualization, data curation, formal analysis. **Shiming Chen**: Formal analysis. **Xunyuan Wu**: Data curation, formal analysis. **Jingjing Zhong**: Data curation, formal analysis. **Meimei Wu**: Data curation, critical revision. **Miaomiao Huo**: Data curation, funding acquisition. All authors read and approved the manuscript.

## CONFLICT OF INTEREST STATEMENT

The authors declared no conflict of interest.

## Data Availability

The data sets used and/or analyzed during the current study available from the corresponding author on reasonable request.
